# Cryptotanshinone as a Multi-Target Natural Terpenoid with Bronchodilator Potential: Insights from Integrated In Vitro and In Silico Studies

**DOI:** 10.3390/molecules31122122

**Published:** 2026-06-16

**Authors:** Naima Salem Rodwan, Aysegul Koc Nas, Saliha Aysenur Cam Ozunlu, Fatma Uysal, Muhammet Zahit Celik, Halil Kara, Seyfullah Oktay Arslan

**Affiliations:** Department of Medical Pharmacology, Faculty of Medicine, Ankara Yıldırım Beyazıt University, 06800 Ankara, Turkey; naimarodwan@gmail.com (N.S.R.); a.koc@aybu.edu.tr (A.K.N.); sacam.ozunlu@aybu.edu.tr (S.A.C.O.); fuysal@aybu.edu.tr (F.U.); mzcelik@aybu.edu.tr (M.Z.C.); halilkara@aybu.edu.tr (H.K.)

**Keywords:** cryptotanshinone, airway smooth muscle, muscarinic receptor, calcium channel, nitric oxide, bronchodilator

## Abstract

Asthma is a chronic airway disease characterized by inflammation, bronchial hyperresponsiveness, and airflow limitation, highlighting the need for novel bronchodilator agents. Cryptotanshinone (CT), a bioactive diterpenoid derived from *Salvia miltiorrhiza*, exhibits anti-inflammatory and vasodilatory properties; however, its direct effects on airway smooth muscle remain poorly characterized. This study investigated the bronchodilatory activity of CT and its pharmacological mechanisms. Molecular docking was performed to evaluate potential interactions with M_3_ muscarinic receptors and L-type calcium channels. Functional experiments were conducted using isolated guinea pig tracheal smooth muscle preparations. The relaxant effects of CT (10^−7^–3 × 10^−4^ M) were evaluated against carbachol (1 µM)- and high-K^+^ (80 mM)-induced contractions. Docking predicted favorable binding of CT to the M_3_ receptor and L-type Ca^2+^ channel, with binding energies of −9.854 and −9.951 kcal/mol, respectively. In vitro, CT produced concentration-dependent relaxation of CCh-induced contractions, reaching a maximal effect of 41.9 ± 2.58% at 3 × 10^−4^ M (pEC_50_ = 4.60). CT produced minimal relaxation in high-K^+^-induced contractions, suggesting receptor-mediated rather than non-selective smooth muscle inhibition. CT also produced a parallel rightward shift of the CCh concentration–response curve at 10^−5^ M, whereas a higher concentration (10^−4^ M) altered the maximal contractile response, suggesting concentration-dependent pharmacological effects. Pharmacological inhibition studies indicated the involvement of muscarinic receptor-mediated mechanisms, with additional contributions from calcium channel-related mechanisms and partial involvement of the NO/cGMP pathway, while β_2_-adrenergic signaling and potassium channels were not significantly involved. These findings suggest that CT exerts bronchodilatory effects through the involvement of multiple pharmacological pathways relevant to airway smooth muscle regulation and provide preliminary mechanistic evidence supporting further investigation.

## 1. Introduction

Asthma is a chronic inflammatory respiratory disease characterized by airway hyperresponsiveness, reversible airway obstruction, and recurrent symptoms such as wheezing, chest tightness, and shortness of breath, all of which substantially impair quality of life worldwide [1]. The disease is multifactorial in nature and is associated with airway inflammation and structural remodeling, leading to excessive airway narrowing and smooth muscle hypercontractility. Despite advances in pharmacological therapy, effective long-term control remains challenging for many patients. Current treatments, including β_2_-adrenergic agonists and corticosteroids, are associated with important limitations such as receptor desensitization, reduced therapeutic efficacy, and systemic adverse effects, which may compromise treatment adherence and clinical outcomes [2]. These challenges highlight the need for safer and more effective alternative therapeutic strategies [3,4,5,6].

Cryptotanshinone (CT) is a lipophilic diterpenoid quinone isolated from Salvia miltiorrhiza (Danshen), a medicinal plant that has been extensively used in traditional medicine for the treatment of inflammatory and respiratory-related disorders [7,8,9]. Phytochemical investigations have identified CT as one of the major bioactive constituents of *S. miltiorrhiza*. Previous studies have shown that CT exhibits anti-inflammatory and immunomodulatory properties, which are highly relevant to airway inflammatory diseases. In experimental models of asthma, CT has been reported to attenuate airway inflammation, reduce mucus hypersecretion, and improve pulmonary function. Despite these therapeutic potentials, the direct effect of CT on airway smooth muscle and the underlying pharmacological mechanisms remain insufficiently characterized, particularly in isolated airway tissue models [10,11,12].

Natural products remain a major source of lead compounds in drug discovery, particularly for complex diseases such as asthma, where multi-target pharmacological approaches are often required. Among these, terpenoids have attracted considerable attention due to their structural diversity and broad spectrum of biological activities, including anti-inflammatory and smooth muscle-modulating effects. Recent evidence has further emphasized that plant-derived compounds can exert therapeutic effects through the simultaneous modulation of inflammatory pathways, calcium signaling, oxidative stress, and smooth muscle function [13,14].In particular, accumulating evidence indicates that tanshinone-derived molecules, including cryptotanshinone, possess multi-target regulatory properties potentially relevant to diseases characterized by airway inflammation and smooth muscle dysfunction. Despite their therapeutic potential, a significant translational gap persists between in vitro pharmacological findings and the development of clinically relevant drug candidates. In this context, investigating the mechanistic effects of bioactive terpenoids such as cryptotanshinone on airway smooth muscle may provide important mechanistic insights and support the identification of novel bronchodilator lead compounds [13,15,16,17].

In addition to its bronchodilator potential, cryptotanshinone has been extensively reported to exert potent anti-inflammatory effects through the inhibition of key signaling pathways such as NF-κB and p38 MAPK, leading to reduced production of pro-inflammatory cytokines including TNF-α, IL-6, and IL-1β, as well as attenuation of airway inflammation and remodeling in experimental models [8,18]. Given that airway inflammation is a central component of asthma pathophysiology, these anti-inflammatory properties may complement its direct smooth muscle relaxant effects, suggesting a dual therapeutic potential of cryptotanshinone in inflammatory airway diseases.

Airway narrowing and obstruction in asthma are primarily due to abnormalities in airway smooth muscle (ASM), including enhanced contractility and impaired relaxation. Relaxation of ASM is therefore a key therapeutic goal. Intracellular calcium ([Ca^2+^]_i_) regulation plays a central role in this process, and agonist-induced [Ca^2+^]_i_ responses are amplified in ASM cells from asthmatic individuals [19]. Acetylcholine (ACh)-induced activation of muscarinic M3 receptors increases intracellular calcium levels and promotes smooth muscle contraction [20].

Conversely, β_2_-adrenergic receptors (β_2_-ARs) are G protein-coupled receptors (GPCRs) that couple to Gs proteins and promote airway smooth muscle relaxation. Upon activation, β2-ARs stimulate the Gs protein, which activates adenylyl cyclase, converting cytosolic ATP into cyclic adenosine monophosphate (cAMP). The generated cAMP then binds to the regulatory subunits of protein kinase A (PKA), thereby activating it. Activated PKA then phosphorylates myosin light chain kinase (MLCK), which inhibits its function. This reduces myosin light chain phosphorylation and leads to decreased intracellular Ca^2+^ mobilisation, thereby contributing to ASM relaxation. PKA modulates the activity of large-conductance Ca^2+^-activated K^+^ (BKCa) channels, abundantly expressed in the ASM membrane. Phosphorylation of these channels induces K^+^ efflux and subsequent cell membrane hyperpolarisation, which further contributes to β2-AR-mediated smooth muscle relaxation [19,21].

K^+^ channels, which are widely found in cells, are the most abundant and varied ion channel family in the human genome. They play a role in many physiological functions, such as neurotransmitter release, action potential, and resting membrane potential. The respiratory system contains four major subtypes of potassium channels: calcium-activated (K_Ca_), inwardly rectifying (K_ir_), ATP-sensitive (K_ATP_) and voltage-gated (K_V_) channels [22]. The activation of BK_Ca_ channels plays a crucial role in controlling several physiological functions, including smooth muscle contraction [23]. This mechanism involving Kv channels helps modulate the contractility and relaxation of smooth muscle cells, thereby influencing various physiological processes in the body [24,25,26]. The most prevalent Kir channels expressed in smooth muscle cells belong to the Kir2.x subtype, particularly Kir2.1, which contributes to key K+ conductance pathways in smooth muscle, nerve, endothelial, and epithelial cells [20,27].

The airway epithelium plays an active role in regulating smooth muscle tone by synthesising and releasing relaxing mediators, notably prostaglandins (PGs) and nitric oxide (NO). These endogenous mediators contribute to bronchodilation, and stimulation of adenylyl cyclase by β2-adrenergic receptor agonists can exert a similar effect to that of NO donors in the small airways. PGs, via the cyclooxygenase (COX) pathway, modulate ASM contraction by converting arachidonic acid (AA) to prostaglandin H_2_, the rate-limiting step in this pathway. In vitro animal studies have demonstrated that either epithelial denudation or exposure to the non-selective COX inhibitor indomethacin or NOS inhibitor L-NAME enhances muscarinic agonist-induced contraction in rabbit bronchi and rat trachea [28,29]. 

Muscarinic acetylcholine receptors (M1–M5) are G protein-coupled receptors (GPCRs), all endogenously activated by acetylcholine (ACh). M1, M2, and M3 subtypes are present in human airways. M3 receptors are highly expressed on bronchial smooth muscle cells and submucosal glands, mediating bronchoconstriction and mucus secretion in response to cholinergic stimulation. Pharmacological blockade of M3 receptors prevents ACh-induced smooth muscle contraction, promoting airway relaxation and bronchodilation [20,30].

The objective of this study was to investigate the relaxant effects of cryptotanshinone (CT) on tracheal smooth muscle using isolated guinea pig tracheal preparations, building upon our previous in silico docking analysis. The study aimed to elucidate the contribution of muscarinic receptors, Ca^2+^ and K^+^ channels, and NO–cGMP and cyclooxygenase-related signaling pathways, while also assessing the potential involvement of β_2_-adrenergic mechanisms.

In addition, molecular docking analyses were performed to support the experimental findings and to provide structural insight into potential CT–target interactions. Collectively, the experimental and computational results are expected to provide novel insights into the bronchodilator mechanisms of CT, potentially informing the development of new therapeutic strategies for respiratory diseases and laying the foundation for future pharmacological studies on plant-derived terpenoids.

## 2. Results

### 2.1. Molecular Docking

Calcium channel blockers and M3 muscarinic receptor antagonists play an important role in current therapeutic strategies for the management of airway bronchoconstriction. Accordingly, these proteins were selected as potential molecular targets to investigate the interaction of cryptotanshinone (CT).

The results of the molecular docking analysis are summarized in Table 1, where binding affinities are expressed as docking scores (kcal/mol). Lower docking scores indicate stronger predicted ligand–receptor interactions.

The values reported in Table 1 represent mean ± SD values calculated from representative docking clusters, whereas the docking values discussed in the text refer to the best-ranked ligand conformations identified during the docking procedure.

The reference compounds levamlodipine and tiotropium exhibited docking scores of −7.255 kcal/mol and −9.312 kcal/mol, respectively. Notably, CT demonstrated more favorable docking scores against the selected targets, suggesting a greater predicted interaction potential compared with the reference molecules.

**Figure 1:**
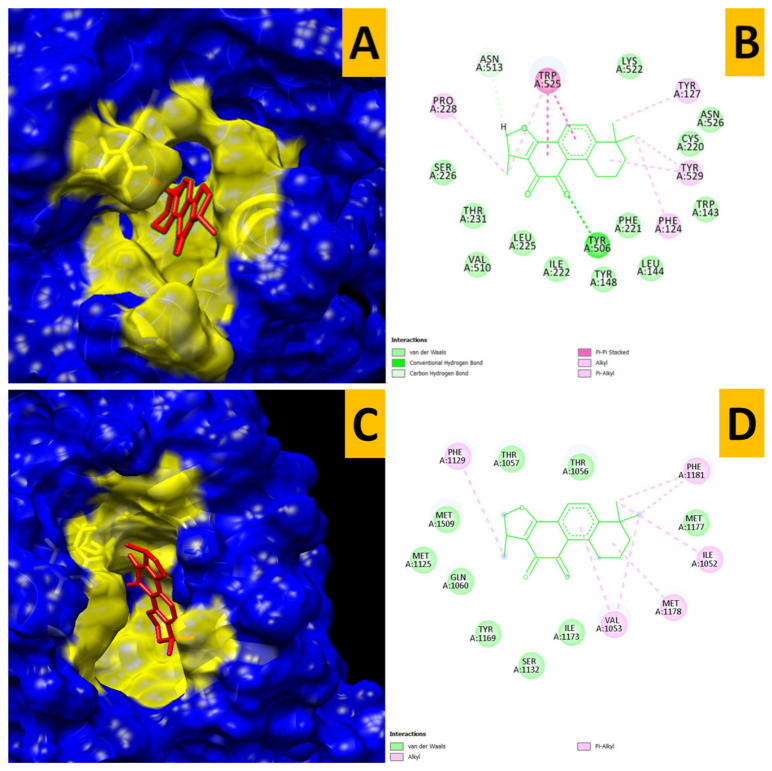
Three-dimensional (3D) molecular docking representations of cryptotanshinone (CT) bound to the active sites of the M3 muscarinic receptor (PDB ID: 4U15) and L-type calcium channel (PDB ID: 8WE8). Panels (**A**,**C**) show the binding poses of CT within the receptor binding pockets with interacting residues highlighted, while panels (**B**,**D**) present the corresponding two-dimensional (2D) interaction diagrams illustrating ligand–receptor interactions.

The predicted binding poses of CT within the active sites of the target proteins are illustrated in Figure 1. Two-dimensional interaction diagrams generated using Discovery Studio Visualizer revealed amino acid residues involved in ligand binding and the interaction types formed between CT and the receptors, including carbon–hydrogen bonds, alkyl interactions, van der Waals interactions, and hydrogen bonds. Previous studies have suggested that hydrophobic and hydrogen-bond interactions contribute substantially to ligand stability, receptor occupancy, and binding affinity, whereas interactions involving aromatic and polar residues may influence ligand–receptor recognition and downstream functional responses [31,32]. Moreover, modulation of calcium signaling pathways has been reported as an important mechanism underlying the smooth muscle regulatory effects of tanshinone-derived compounds [9,33].

Based on these in silico findings suggesting potential interactions of CT with muscarinic receptors and calcium channels, subsequent in vitro experiments were performed to evaluate the bronchodilatory effects of CT on isolated tracheal smooth muscle.

### 2.2. Effect of CT on Isolated Tracheal Rings

**Figure 2:**
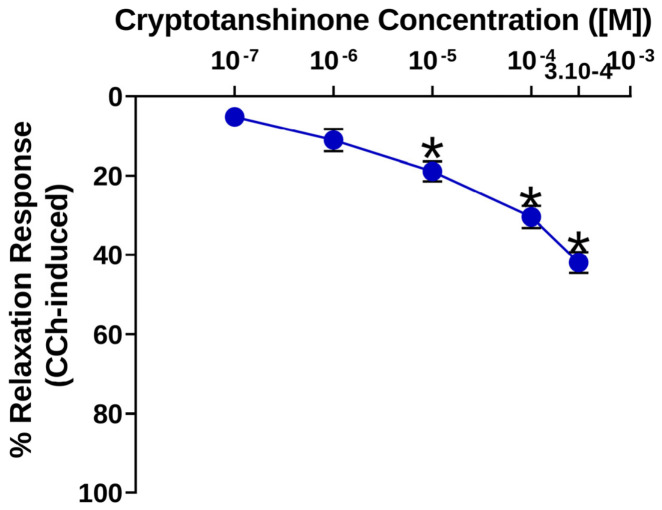
Concentration-dependent relaxant effect of cryptotanshinone (CT) on carbachol (CCh)-induced contractions in isolated tracheal rings. Tracheal rings were precontracted with CCh (1 μM) and increasing concentrations of CT (10^−7^–3 × 10^−4^ M) were applied cumulatively. Values are presented as mean ± SEM (*n* = 8, tissues derived from different animals). * *p* < 0.05 vs. CCh control.

CT (1 × 10^−7^–3 × 10^−4^ M) exhibited a concentration-dependent relaxant effect on tracheal strips precontracted with CCh (1 µM). CT at concentrations of 10^−4^ and 3 × 10^−4^ M reduced CCh-induced contraction responses by 30.4 ± 2.73% and 41.9 ± 2.58%, respectively (Figure 2). The maximal relaxant effect of CT against CCh-induced contraction (Emax = 41.9 ± 2.58%) was observed at 3 × 10^−4^ M, with an estimated pEC50 value of 4.60 for CT-induced relaxation of CCh-precontracted tracheal tissues.

**Figure 3:**
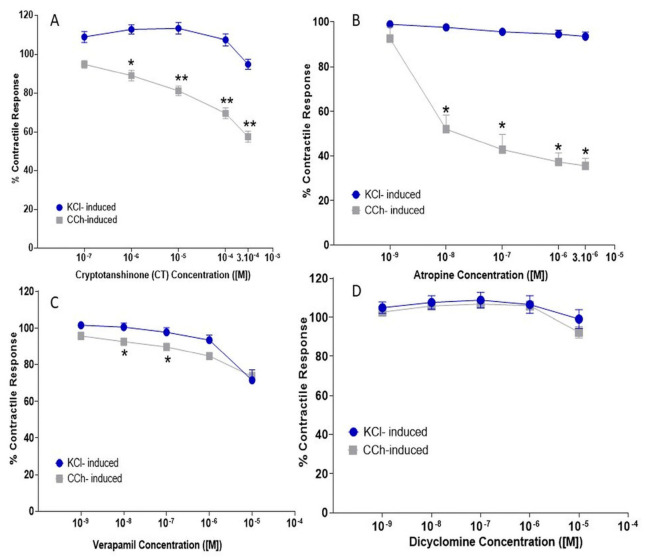
Concentration–response curves showing the inhibitory effects of (**A**) cryptotanshinone (CT), (**B**) atropine, (**C**) verapamil, and (**D**) dicyclomine on contractions induced by KCl (80 mM) and carbachol (CCh, 1 μM) in isolated guinea pig tracheal preparations. Values are presented as mean ± SEM (*n* = 6). * *p* < 0.05, ** *p* < 0.01 compared with the corresponding CCh- or KCl-induced control response.

In contrast, CT produced only a weak relaxation in tracheal rings precontracted with high K^+^ (80 mM). At 3 × 10^−4^ M, CT reduced K^+^-induced contraction by 5.1 ± 2.53%, which was significantly lower than its effect on CCh-induced contractions (Figure 3A).

Atropine (10^−9^–3 × 10^−6^ M) produced a marked concentration-dependent relaxation of CCh-induced contractions. Atropine at 10^−6^ and 3 × 10^−6^ M reduced contraction responses by 62.8 ± 4.03% and 64.5 ± 3.35%, respectively, with minimal effects on high-K^+^-induced contractions (Figure 3B).

Verapamil (10^−9^–10^−5^ M) also produced concentration-dependent relaxation of CCh-induced contractions. The relaxant responses were more pronounced in CCh-precontracted tissues compared with those contracted with high K^+^ (Figure 3C).

Dicyclomine (10^−9^–10^−5^ M) reduced CCh-induced contraction responses in a concentration-dependent manner; however, these effects did not reach statistical significance (*p* > 0.05). (Figure 3D).

The greater inhibitory effect of CT on CCh-induced contractions compared with high-K^+^-induced responses suggests that CT preferentially interferes with receptor-dependent contractile mechanisms rather than acting as a non-selective smooth muscle relaxant.

### 2.3. Effect of Muscarinic Receptor Activity on Isolated Tracheal Rings

**Figure 4:**
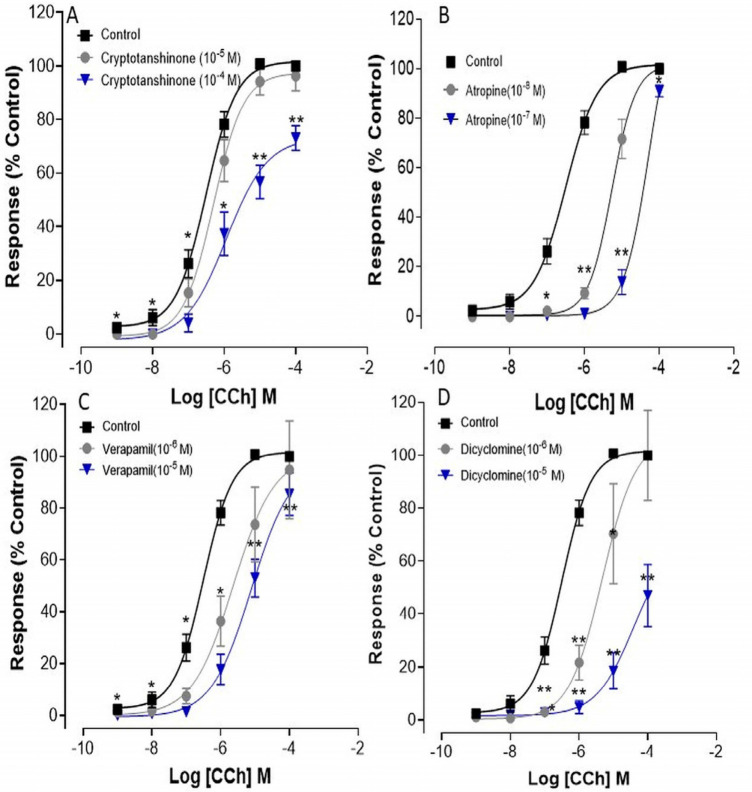
Concentration–response curves of carbachol (CCh) in the absence (control) and presence of increasing concentrations of (**A**) CT, (**B**) atropine, (**C**) verapamil and (**D**) dicyclomine in isolated guinea-pig tracheal preparations. Tissues were preincubated with CT (10^−5^ and 10^−4^ M), atropine (10^−8^ and 10^−7^ M), verapamil (10^−6^ and 10^−5^ M), or dicyclomine (10^−6^ and 10^−5^ M) before cumulative addition of CCh (10^−9^–1 × 10^−4^ M). Values are presented as mean ± SEM (*n* = 6, tissues derived from different animals). * *p* < 0.05, ** *p* < 0.01 compared with the corresponding control concentration–response curve.

In the absence of CT, cumulative administration of CCh produced concentration-dependent contractions reaching a maximal response of 100%. Pre-incubation with CT (10^−5^ M) induced a parallel rightward shift of the CCh concentration–response curve without affecting the maximal response, indicating the presence of a surmountable antagonistic component. However, at a higher CT concentration (10^−4^ M), the maximal contractile response to CCh was reduced to 73%, indicating partial suppression of the contractile response (Figure 4A).

Similarly, pre-incubation with dicyclomine (10^−6^ and 10^−5^ M) produced a rightward shift of the CCh concentration–response curves with partial suppression of maximal responses (Figure 4D). Verapamil (10^−6^ and 10^−5^ M) also induced a rightward shift of the CCh curves without reducing maximal contraction (Figure 4C). In contrast, atropine (10^−8^ and 10^−7^ M) caused a parallel rightward shift of the CCh curves without affecting maximal responses (Figure 4B).

These results suggest that CT exerts a functional antagonistic effect on muscarinic receptor-mediated contractions.

### 2.4. Effects of NOS/cGMP and COX2-PGE2 Signalling Pathways on Isolated Tracheal Rings

In control tracheal strips, CT (1 × 10^−7^–3 × 10^−4^ M) produced concentration-dependent relaxation of CCh-induced contractions with a maximal effect (Emax) of 41.9 ± 2.58%.

In tracheal strips incubated with the nitric oxide synthase (NOS) inhibitor L-NAME (10^−4^ M), CT reduced CCh-induced contraction responses in a concentration-dependent manner; however, the magnitude of relaxation was significantly lower than that observed in the control group, with a maximal effect (Emax) of 32.3 ± 1.66% at 3 × 10^−4^ M.

Similarly, in the presence of the soluble guanylate cyclase inhibitor ODQ (10 µM), CT at concentrations of 10^−5^ and 10^−4^ M significantly reduced CCh-induced contractions, although these relaxant effects were attenuated compared with the control group.

**Figure 5:**
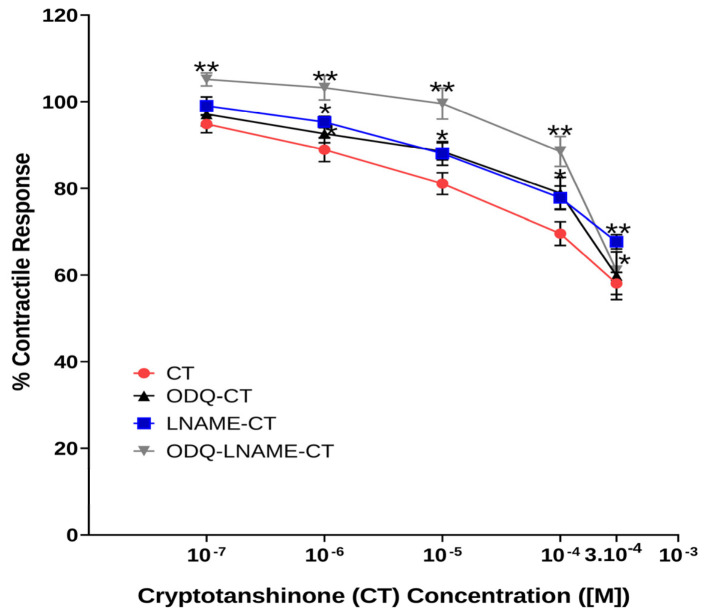
Effects of CT in the absence and presence of NOS/cGMP pathway inhibitors on CCh (1 μM)-induced contractions in isolated tracheal rings. Tissues were preincubated with CT (10^−7^–3 × 10^−4^ M) alone or in the presence of ODQ (10 μM), L-NAME (100 μM), or their combination before cumulative addition of CT. Values are presented as mean ± SEM (*n* = 6, tissues derived from different animals). * *p* < 0.05, ** *p* < 0.01 compared with the CT group at the same concentration.

Combined inhibition of the NOS/cGMP pathway using L-NAME and ODQ further diminished the relaxant effects of CT across all tested concentrations (Figure 5).

In tracheal strips pre-incubated with the cyclooxygenase inhibitor indomethacin (10^−5^ M), CT (1 × 10^−7^–3 × 10^−4^ M) did not produce a significant change in CCh (1 µM)-induced contraction responses compared with the control group.

Collectively, these results indicate that inhibition of the NOS/cGMP pathway attenuates CT-induced relaxation, suggesting a partial contribution of nitric oxide-dependent mechanisms.

### 2.5. Effect of Calcium Channels (Ca^2+^) on Isolated Tracheal Rings

To evaluate the involvement of extracellular calcium influx in CT-induced relaxation, contractile responses to cumulative CaCl_2_ administration were assessed in isolated tracheal rings.

In control tissues, cumulative CaCl_2_ administration produced concentration-dependent contractions reaching a maximal response (Emax) of 129.77 ± 9.48%.

**Figure 6:**
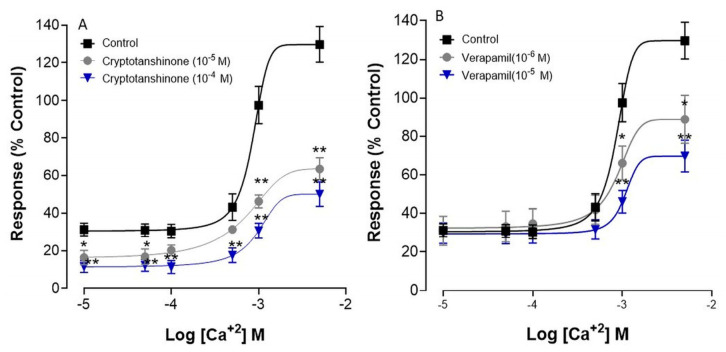
Concentration–response curves of Ca^2+^ in the absence (control) and presence of increasing concentrations of (**A**) CT and (**B**) verapamil in isolated guinea pig tracheal preparations. Values are presented as mean ± SEM (*n* = 6, tissues derived from different animals). * *p* < 0.05, ** *p* < 0.01 compared with the corresponding control response.

Pre-incubation with CT (10^−5^ and 10^−4^ M) significantly reduced CaCl_2_-induced contractile responses across the tested concentration range. The maximal contractions were decreased to 63.56 ± 6.03% at 10^−5^ M and 49.99 ± 6.48% at 10^−4^ M, respectively (Figure 6A).

Similarly, verapamil (10^−6^ and 10^−5^ M) significantly attenuated CaCl_2_-induced contractions, with maximal responses reduced to 88.94 ± 12.49% and 69.77 ± 8.25%, respectively, consistent with inhibition of calcium influx (Figure 6B).

### 2.6. Effect of β2-Adrenergic/cAMP Activity on Isolated Tracheal Rings

In tracheal strips pre-incubated with propranolol (1 µM), CT (1 × 10^−7^–3 × 10^−4^ M) reduced CCh-induced contractions in a concentration-dependent manner; however, these effects were not significantly different from those observed with CT alone.

In the presence of the adenylate cyclase inhibitor SQ22,536 (100 µM), low concentrations of CT slightly increased CCh-induced contractions, although this effect did not reach statistical significance (*p* > 0.05). Combined incubation with propranolol and SQ22,536 also did not significantly modify CT-induced relaxation.

These findings indicate that β_2_-adrenergic receptor/cAMP signalling is unlikely to play a major role in the relaxant effect of CT on tracheal smooth muscle.

### 2.7. Effects of Potassium (K+) Channel Activity on Isolated Tracheal Rings

Since potassium channel activation has been reported to contribute to airway smooth muscle relaxation, the potential involvement of K^+^ channels in CT-induced bronchodilation was also evaluated.

CT (1 × 10^−7^–3 × 10^−4^ M) did not significantly modify CCh-induced contraction responses in tracheal strips pre-incubated with different K^+^ channel blockers, including TEA (100 µM), 4-aminopyridine (100 µM), and glibenclamide (1 µM).

Similarly, CT-induced relaxant responses were not significantly different from those observed in the absence of these inhibitors.

These findings suggest that potassium channel activation does not appear to play a significant role in CT-induced relaxation in tracheal smooth muscle.

## 3. Discussion

Airway remodelling and the associated structural alterations are central features of asthma pathophysiology and are closely linked to airway smooth muscle (ASM) dysfunction. These changes contribute to exaggerated airway narrowing and hyperresponsiveness, highlighting the importance of directly targeting ASM tone in bronchodilator research. In this context, the present study investigated the relaxant effect of cryptotanshinone on isolated guinea pig tracheal smooth muscle and explored the pharmacological mechanisms that may underlie its bronchodilator activity.

Previous studies have shown that cryptotanshinone reduces airway hyperresponsiveness and improves pulmonary function in experimental asthma models, effects that have largely been attributed to its anti-inflammatory and immunomodulatory actions, including modulation of NF-κB signaling and p38 MAPK activation [10,12,18]. In addition to these effects, cryptotanshinone and structurally related diterpenoids derived from *Salvia miltiorrhiza*, such as tanshinone IIA, have been reported to induce concentration-dependent relaxation in vascular smooth muscle preparations. Based on these findings, we hypothesised that cryptotanshinone may also exert a direct relaxant effect on airway smooth muscle.

Molecular docking simulations suggested that cryptotanshinone (CT) exhibited favourable binding affinities toward both the M3 muscarinic receptor and the L-type voltage-dependent calcium channel (L-VDCC), indicating a potential role in the modulation of airway smooth muscle tone. The best-ranked docking poses demonstrated binding energies of −9.854 kcal/mol for the M3 receptor and −9.951 kcal/mol for the L-type Ca^2+^ channel, indicating energetically stable ligand–target interactions.

The molecular docking results demonstrated that the high-scoring binding regions of our ligand are parallel with those reported in the literature, supporting our computational predictions. Specifically, for the M3 muscarinic receptor (PDB ID: 4U15), our compound established interactions with residues TYR148, THR231, TYR506, and TYR529 [10,34]. These findings are in strong agreement with a previous study by Lin et al., which highlighted that interactions with these specific amino acids are critical for effective ligand binding within the active site [35]. Similarly, for the L-type voltage-dependent calcium channel (PDB ID: 8WE8), our ligand shared a common binding pocket and exhibited significant interactions with residues THR1056, THR1057, GLN1060, SER1132, TYR1169, and MET1509 [9]. Consistent with the mechanistic insights provided by Logan et al., interactions with these key residues are considered crucial for the functional modulation of the receptor [36]. This comparative analysis confirms that our ligand targets the commonly reported pharmacophore-related features of both receptors, further supporting the reliability of our docking outcomes.

Taken together, these in silico findings provide supportive, although not definitive, evidence that CT may interact with both muscarinic receptors and voltage-dependent calcium channels, thereby offering a plausible molecular framework for the potential bronchodilatory effects of CT on airway smooth muscle. Based on these observations, subsequent in vitro experiments were performed to evaluate the functional relaxant effects of CT in isolated tracheal smooth muscle preparations.

Previous studies have suggested that hydrophobic interactions and hydrogen-bond formation represent important determinants of ligand stability, receptor occupancy, and binding affinity, whereas interactions involving aromatic and polar residues may influence ligand–receptor recognition and downstream functional responses [31,32]. Furthermore, modulation of calcium signaling pathways has been identified as an important mechanism underlying the smooth muscle regulatory effects of tanshinone-derived compounds [9,33]. Therefore, the interaction profile observed for CT may provide a structural basis supporting its experimentally observed bronchodilator activity.

The present findings extend observations from our previous exploration in silico study, which suggested the bronchodilator potential of plant-derived compounds including cryptotanshinone [34]. While the previous work provided preliminary computational evidence regarding potential target interactions, the current study expands upon these observations through functional pharmacological experiments using isolated guinea pig tracheal smooth muscle and provides additional mechanistic insights into the bronchodilatory effects of CT.

Airway smooth muscle tone is tightly regulated by multiple signaling pathways, including muscarinic and β_2_-adrenergic receptors, ion channels, and nitric oxide-dependent mechanisms [24,37,38,39].To clarify the pathways contributing to cryptotanshinone (CT)-induced relaxation, a series of pharmacological interventions targeting these regulatory systems were employed in the guinea pig tracheal smooth muscle model.

The bronchodilator effect of CT and its mechanisms of action have not been fully explored in vitro or in vivo. Tanshinone IIA has been shown to induce smooth muscle relaxation by inhibiting voltage-dependent calcium channels and activating ATP-sensitive potassium channels [40]. In the present study, for the first time, a concentration-dependent relaxant effect of CT on CCh-induced contractions was demonstrated in isolated guinea pig tracheal smooth muscle.

High-KCl-induced contractions primarily reflect membrane depolarization and extracellular Ca^2+^ influx through voltage-dependent calcium channels, whereas carbachol induces contraction mainly via muscarinic receptor activation involving both extracellular Ca^2+^ entry and intracellular Ca^2+^ release [41]. The greater inhibitory effect of CT on CCh-induced contractions compared with high-KCl-induced responses suggests a preferential interference with receptor-dependent contractile mechanisms, including muscarinic receptor activation and downstream calcium signaling pathways. This observation also indicates that CT does not act as a non-selective smooth muscle relaxant but rather preferentially modulates receptor-mediated contractile signaling.

Atropine, as expected, selectively inhibited CCh-induced contractions, confirming the involvement of muscarinic receptors. Both atropine and CT caused a parallel rightward shift of the CCh concentration–response curve, suggesting a functional antagonistic effect at muscarinic receptors. CT (10^−5^ M) produced a parallel rightward shift of the CCh concentration–response curve without reducing the maximal response, suggesting the presence of a surmountable antagonistic component. However, at a higher concentration (10^−4^ M), CT reduced the maximal contractile response to CCh, indicating a concentration-dependent alteration in the contractile response profile. The observed concentration–response curve patterns may suggest the coexistence of distinct antagonistic behaviors across different concentration ranges; however, these findings should be interpreted as qualitative pharmacological observations rather than definitive mechanistic characterization [42].

Pre-incubation with dicyclomine (10^−6^ M and 10^−5^ M) similarly produced a parallel rightward shift in the CCh response curve, further supporting muscarinic receptor antagonism. However, although dicyclomine did not produce statistically significant relaxation in the direct relaxation experiments, its effects became more evident in cumulative concentration–response analyses, suggesting that different experimental paradigms may reveal distinct pharmacological characteristics. In contrast, verapamil produced a significant relaxation of CCh-induced contractions accompanied by a non-parallel rightward shift and suppression of the maximal response, indicating a mechanism consistent with voltage-dependent calcium channel inhibition rather than direct muscarinic receptor antagonism.

Pharmacological analyses further indicated that the examined potassium channel subtypes do not appear to play a significant role in CT-induced relaxation under the present experimental conditions. Neither the non-selective potassium channel blocker TEA, the voltage-dependent potassium channel inhibitor 4-aminopyridine, nor the ATP-sensitive potassium channel blocker glibenclamide significantly altered the relaxant effect of CT. In contrast, evaluation of calcium-dependent mechanisms revealed that CT significantly inhibited CaCl_2_-induced contractions in depolarized tissues, an effect consistent with suppression of voltage-dependent calcium influx. The similarity between these responses and those observed with verapamil under identical experimental conditions supports the involvement of calcium channel inhibition. However, as this effect became evident only at higher concentrations of CT, it is likely to represent a complementary rather than primary mechanism.

Further mechanistic investigations indicated that the β_2_-adrenergic receptor/cAMP signaling pathway does not contribute significantly to the bronchodilator effect of CT. Pre-incubation with the β2-adrenergic receptor antagonist propranolol and the adenylate cyclase inhibitor SQ22,536 did not significantly alter CT-induced relaxation of CCh-contracted tissues, indicating that CT exerts its effects independently of β_2_-adrenergic receptor activation and cAMP signaling.

In the present study, under blockade conditions with the NO synthase inhibitor L-NAME and the soluble guanylate cyclase inhibitor ODQ, a statistically significant reduction in CT-induced relaxation was observed. This suggests that the tracheal smooth muscle relaxation caused by CT may be partially mediated through airway epithelium- and NOS-derived NO production and the downstream cGMP signaling pathway. Although this pathway does not appear to represent the primary mechanism of action, its involvement is consistent with previous reports demonstrating NO-dependent vasorelaxant effects of cryptotanshinone and related tanshinones in vascular smooth muscle [9,43]. Collectively, our findings suggest that the activation of the NO/cGMP pathway may function as an adjunct mechanism supporting CT-induced relaxation of airway smooth muscle.

In contrast, blockade of the COX-2/PGE_2_ pathway using indomethacin did not significantly affect CT-induced relaxation, indicating a minimal contribution of prostaglandin-dependent mechanisms.

Consistent with these findings, our combined in silico docking analysis and functional in vitro experiments indicate that muscarinic receptor antagonism represents the primary mechanism underlying CT-induced airway relaxation, whereas inhibition of voltage-dependent calcium influx appears to contribute as a secondary pathway. Furthermore, the reduction of relaxation following NOS/cGMP blocking suggests that nitric oxide-dependent signaling plays a role as an additional mechanism. These pathways are not mutually exclusive, and their potential functional convergence may contribute to the overall pharmacological profile of cryptotanshinone. The involvement of multiple pharmacological pathways may be relevant for the modulation of airway hyperresponsiveness, which is regulated by multiple interacting signaling pathways in airway smooth muscle.

Several limitations of the present study should be acknowledged. The bronchodilatory effects of cryptotanshinone were evaluated using an isolated guinea pig tracheal smooth muscle model, which is well-suited for mechanism-based pharmacological investigations focused on direct functional responses. However, molecular docking was employed as a supportive in silico approach and therefore does not provide direct experimental evidence of ligand–target binding or functional modulation. While functional pharmacological interventions were used to infer pathway involvement, direct molecular or electrophysiological validation of these interactions was beyond the scope of the present study. Since molecular docking was utilized strictly as supportive evidence, advanced computational analyses such as molecular dynamics simulations and ab initio ligand optimization were not performed, representing an additional limitation of the study. Future studies incorporating advanced computational validation approaches together with in vivo pharmacodynamic and safety evaluations in experimental asthma models would be valuable to further define the translational relevance of cryptotanshinone. In addition, receptor subtype selectivity was not directly evaluated in the present study. Therefore, the specific contribution of M3 receptors should be interpreted cautiously and warrants further investigation.

Beyond its direct bronchodilatory effects, the potential anti-inflammatory properties of cryptotanshinone may further enhance its therapeutic relevance in airway diseases. Asthma and other obstructive pulmonary diseases are characterized not only by airway smooth muscle hyperresponsiveness but also by chronic inflammation involving multiple immune mediators. Previous studies have demonstrated that cryptotanshinone suppresses key inflammatory signaling pathways such as NF-κB and p38 MAPK, reduces pro-inflammatory cytokine production, and attenuates airway inflammation and remodeling in experimental models [8,12,18]. Therefore, the combined bronchodilator and anti-inflammatory actions of cryptotanshinone may provide a synergistic therapeutic advantage by targeting both functional and inflammatory components of airway dysfunction.

From a translational perspective, the present findings suggest that cryptotanshinone may represent a potential natural lead scaffold for further investigation in bronchodilator drug discovery. The observed muscarinic receptor antagonism, supported by the parallel rightward shift of the CCh concentration–response curve, is consistent with the mechanism of clinically established bronchodilators such as atropine and tiotropium. In addition, CT demonstrated modulatory effects involving calcium influx and NO/cGMP signaling pathways under the present experimental conditions. The involvement of multiple pharmacological pathways may be relevant in airway hyperresponsiveness, a condition regulated by complex and overlapping signaling mechanisms. Although CT exhibited relaxant activity at concentrations higher than those commonly reported for clinically established bronchodilators such as tiotropium and β_2_-agonists, direct potency comparisons should be interpreted cautiously because the present study was designed to investigate both the functional relaxant effects and the underlying pharmacological mechanisms of CT rather than direct comparison with optimized therapeutic bronchodilators [13,44,45].

In summary, the present study demonstrates that the relaxant effects of cryptotanshinone on isolated tracheal smooth muscle are mediated primarily through muscarinic receptor antagonism, particularly under cholinergic stimulation. In addition, inhibition of voltage-dependent calcium influx and downstream calcium mobilization contributes to CT-induced relaxation at higher concentrations. Partial involvement of the NO/cGMP signaling pathway was also observed, whereas β_2_-adrenergic receptors and potassium channels showed minimal or no contribution. Overall, these findings suggest that cryptotanshinone exerts bronchodilatory effects through the involvement of multiple pharmacological pathways, including muscarinic receptor-mediated mechanisms, calcium signaling, and NO/cGMP-related pathways under the present experimental conditions. Additional in vivo and translational studies are required to further clarify its therapeutic potential and clinical relevance.

## 4. Materials and Methods

### 4.1. Animals

Twenty male adult guinea pigs (4–6 months old, 300–500 g) were used in this study. All experimental procedures were approved by the Animal Experiments Ethics Committee of the University of Health Sciences, Education and Research Hospital (22 May 2024-779) and were conducted in accordance with institutional and national guidelines for the care and use of laboratory animals. All experimental processes were carried out in Ankara Yıldırım Beyazıt University, Department of Medical Pharmacology Research Laboratory. The animals were fed with standard laboratory food and tap water ad libitum under controlled-temperature (20 ± 2 °C) conditions and a 12-h light–dark cycle. For all experiments, *n* refers to the number of independent tracheal tissue preparations included in each experimental protocol.

### 4.2. Chemicals

Cryptotanshinone (≥95% purity, Cayman Chemical, Ann Arbor, MI, USA) was used in this study. According to the Certificate of Analysis (Item No. 16987, batch 0610035), the compound was characterized by HPLC (98.3% purity), TLC (100%), IR, NMR, mass spectrometry (m/z 297.1 [MH^+^]), and melting point determination (195–197 °C, dec.), confirming its identity and purity.

Dicyclomine, ODQ, verapamiland propranolol were obtained from Cayman Chemical (Ann Arbor, MI, USA). L-NAME, carbachol, atropine, tetraethylammonium, 4-aminopyridine, glibenclamide, indomethacin, iberiotoxin, and Ethylenediaminetetraacetic acid disodium salt (EDTA-Na_2_) were purchased from Sigma-Aldrich (St. Louis, MO, USA).

Carbachol, 4-aminopyridine, iberiotoxin, dicyclomine, tetraethylammonium, and EDTA-Na_2_ were dissolved in distilled water. Glibenclamide, cryptotanshinone, verapamil, L-NAME, ODQ, propranolol, and indomethacin were dissolved in DMSO and stored at −20 °C until use. On the day of the experiments, all drugs were freshly diluted in Krebs solution to the required final concentration. The Krebs–Henseleit solution used in the bath solution with following composition (mM): NaCl 118; KCl 4.7; NaHCO_3_ 25; NaH_2_PO_4_-2H_2_O 0.9; CaCl_2_-2H_2_O 1.26; MgCl-6H_2_O 0.5; and glucose monohydrate 11. The composition of the Krebs–Henseleit solutionof 80 mM KCl (mM) was NaCl (42.7 mM), KCl (80.0 mM), NaHCO_3_ (25 mM), NaH_2_PO_4_-2H_2_O (0.9 mM), CaCl_2_-2H_2_O (1.26 mM), MgCl-6H_2_O (0.5 mM) and Glucose monohydrate (11 mM) in distilled water. CaCl_2_.2H_2_O, which was to be added to Krebs solution, was weighed in a separate beaker and the other chemicals were weighed and dissolved in another beaker with some distilled water. Then CaCl_2_.2H_2_O solution was slowly added to the solution of other chemicals and added to 2 L.

### 4.3. In Silico Experiments

#### 4.3.1. Protein Selection and Preparation

The X-ray crystallographic structures of the target proteins—namely the M3 muscarinic receptor (PDB ID: 4U15; resolution: 2.80 Å) and the L-type voltage-dependent calcium channel (L-VDCC, PDB ID: 8WE8; resolution: 2.90 Å)—were retrieved from the Protein Data Bank (https://www.rcsb.org). The three-dimensional structures were imported into UCSF Chimera and prepared using the DockPrep tool (Chimera 1.19).

During the preparation process, water molecules were removed, polar hydrogens were added, and Gasteiger charges were calculated. This standard protocol was deemed sufficient, and therefore no separate energy minimisation was required. To simplify the system and enhance docking accuracy, all chains and non-essential residues—excluding chain A—were removed. This step was undertaken to eliminate structural components—such as non-essential chains and residues—that may not be biologically relevant and could potentially interfere with docking analyses. In X-ray structures, such components can arise due to crystal packing artifacts, while in cryo-EM structures, they may result from modeling ambiguities or low-resolution regions. Therefore, only chain A was retained to ensure that the docking process focused on the most biologically relevant and structurally accurate region. Selecting a specific chain, particularly when the docking is focused on a known active site or protein interface, ensures that the process targets the biologically relevant region.

The final structures were converted into the PDBQT format required for docking. Similarly, the 3D structure of CT was downloaded from PubChem in SDF format and underwent an analogous preparation process.

#### 4.3.2. Molecular Docking

An insilico docking approach was employed to investigate ligand–receptor interactions. Molecular docking simulations were performed using AutoDock Vina (1.2.3), integrated into the UCSF Chimera environment. For each target protein, the docking grid was defined based on the coordinates of the co-crystallized ligand present in the original PDB structure.

The grid box dimensions were set to 24 × 24 × 24 Å for the M3 receptor (PDB ID: 4U15) and 50 × 50 × 50 Å for the L-VDCC (PDB ID: 8WE8). The grid centers were defined at the coordinates of (x = 47.26, y = 92.48, z = 55.15) and (x = 156.42, y = 162.23, z = 150.17), respectively. All docking parameters were left at their default settings, except that all rotatable bonds in the ligand were allowed full flexibility, while the receptor was treated as rigid. Docking simulations were executed with an exhaustiveness value of 8.

The final docking poses were evaluated not only by their binding affinity scores but also by their cluster populations. The conformation with the highest docking score residing within the most populated cluster was selected as the representative binding mode for further analysis. Visualization of docked complexes was performed using Discovery Studio Visualizer 3.0, and two-dimensional interaction diagrams were generated to illustrate the binding modes.

#### 4.3.3. Validation of Docking Protocol

To validate the docking protocol and confirm the accuracy of the predicted binding sites, redocking simulations were performed on both target proteins using their respective native ligands: tiotropium for 4U15 and levamlodipine for 8WE8. Each ligand was redocked into its original binding site using the same grid parameters described above.

The Root-Mean-Square Deviation (RMSD) values of the docked ligand poses—calculated across 10 conformations for 8WE8 and 9 conformations for 4U15—were found to be 2.07 Å and 0.827 Å, respectively. Although the RMSD value for 8WE8 slightly exceeds the commonly accepted threshold of 2 Å, it may still be considered acceptable but should be interpreted cautiously, as it represents borderline agreement between the predicted and reference binding poses and may reflect ligand flexibility as well as limitations associated with rigid docking approaches. In contrast, the RMSD value for 4U15 was well within the acceptable range, supporting the reliability of the docking protocol for this target. Overall, these findings provide supportive evidence for the reliability of the docking protocol.

### 4.4. In Vitro Experiments

#### 4.4.1. Isolation of Tracheal Smooth Muscle from Guinea Pigs

Guinea pigs were anaesthetised with ketamine/xylazine (40 mg/kg/5 mg/kg, i.p.) and sacrificed. The thorax was opened to expose the tracheal tissues, and then the tissues were quickly placed in Krebs solution. The tissues were freed from surrounding fat and connective tissues before being divided into 6–8 rings, each 2–3 mm long [46]. The temperature of the isolated organ bath was maintained at 37°C, the pH of the solution was maintained at 7.4, and the tissues placed in it were ventilated with 95% O_2_ and 5% CO_2_.

#### 4.4.2. Isolated Organ Bath Experiments

At the start of the experiments, 1 g of pretension was applied to the tissues, which were then washed every 15 min and allowed to equilibrate for one hour. After this resting and equilibration period, we evaluated the viability of the strips with 80 mM potassium chloride (KCl) and tested their contractile strength.Then, the tissues were washed every 15 min and rested under 1 g tension for 60 min to start each new protocol.

Tissues were mounted in a 10 mL organ bath containing Krebs–Henseleit solution with one end connected to a tissue sling and the other end connected to a force transducer. Isometric smooth muscle contraction and relaxation were recorded by transducers (BIOPAC, MP36 System Inc.).

To investigate the relaxant mechanisms, the tissues were pre-incubated with various antagonists and inhibitors for 20 min before the addition of the contractile agent, and then the effect of CT was examined.

Guinea pig isolated tracheal strips were allowed to equilibrate for one hour before any test drug was added. In each tissue, the effect of only one substance on control responses was analysed. In another group of tissues, the effect of the solvents of the substances used in the experiments on the contraction responses was examined.

#### 4.4.3. Experimental Groups

##### Experimental Design and Tissue Allocation

Tracheal rings obtained from twenty guinea pigs were distributed across different experimental protocols. From each animal, 6–8 tracheal rings were prepared and allocated to separate experimental groups, such that tracheal rings from the same animal were not used within the same protocol. Each experimental condition was therefore performed using tissues derived from different animals.

##### Evaluation of the Effect of Cryptotanshinone on Isolated Tracheal Rings

The relaxant effect of CT (1 × 10^−7^–3 × 10^−4^M) was evaluated on contraction responses induced by CCh (1 µM) or KCl (80 mM) in isolated tracheal rings with dicyclomine (1 × 10^−9^ –1 × 10^−5^M) used as a positive control. Increasing concentrations of atropine (1 × 10^−9^–3 × 10^−6^ M) and verapamil (1 × 10^−9^–1 × 10^−5^M) were applied to CCh-induced contraction to determine effective doses [47,48]. Concentration–response curves were generated with CT added cumulatively every 10 min, and results were expressed as a percentage of contraction responses.

Percent relaxation for each concentration of cumulative doses of CT was calculated according to the maximum contraction with KCl or CCh. Concentration–response curves were plotted using concentration on the *X*-axis and percentage relaxation on the *Y*-axis. At the end of the protocols, the tissues were washed for 60 min to allow the substances to completely lose their effect. Each experiment was performed in a different animal tissue, and the effect of only one drug was analysed.

##### Evaluation of the Effect of Muscarinic Receptor Activity on Isolated Tracheal Rings

To investigate the contribution of muscarinic type anticholinergic activity to the relaxant effects of CT on tracheal smooth muscle, concentration–response curves were obtained by cumulative addition of CCh (1 × 10^−9^–1 × 10^−4^ M) in the presence and absence of CT. As a control group, CCh concentrations were applied cumulatively after pre-incubation with increasing concentrations of atropine (0.01–0.1 µM), dicyclomine (1–10 µM) and verapamil (1–10 µM) [49]. Concentration–response curves were obtained with the effects of CT and atropine, dicyclomine and verapamil on CCh. Relaxation responses were expressed as a percentage of the contraction induced by CCh.

##### Evaluation of the Effects of NOS/cGMP and COX2-PGE2 Signalling Pathways on Isolated Tracheal Rings

The role of nitric oxide- and prostaglandin-related pathways in CT-induced relaxation of tracheal smooth muscle was evaluated using the nitric oxide synthase inhibitor L-NAME (10^−4^ M), the soluble guanylate cyclase inhibitor ODQ (10 μM), and the non-selective cyclooxygenase inhibitor indomethacin (10 μM). Tracheal tissues were precontracted with carbachol (CCh, 1 μM), and cumulative concentrations of CT (1 × 10^−7^–3 × 10^−4^ M) were subsequently added [26]. Relaxation responses were expressed as a percentage of the contraction induced by CCh.

##### Evaluation of the Effect of Calcium Channels (Ca^2+^) on Isolated Tracheal Rings

The Ca^2+^ channel-blocking activity of CT was investigated using isolated tracheal tissues. After stabilisation in normal Krebs solution, the tissues were equilibrated in Ca^2+^-free Krebs solution containing EDTA (0.1 mM) and then incubated for 30 min to remove Ca^2+^ from the tissue. The medium was then replaced with a high-K^+^ (80 mM), Ca^2+^-free Krebs solution, and tissues were further incubated for one hour. Cumulative CaCl_2_-induced contractions were subsequently recorded to establish concentration–response curves. These procedures were repeated in the presence of increasing concentrations of CT (1 × 10^−7^–3 × 10^−4^ M) and compared with verapamil (1 and 10 µM) as a positive control [34]. Concentration-relaxation curves were generated cumulatively.

##### Evaluation of the Effect of B2-Adrenergic/cAMP Activity on Isolated Tracheal Rings

The role of β2-adrenergic receptors in the relaxant effects of CT on tracheal smooth muscle was investigated in the absence and presence of the non-specific β-adrenergic receptor antagonist propranolol (1 µM) and the adenylate cyclase inhibitor SQ22,536 (100 μM) before contraction with CCh (1 µM) [50]. The effect of a cumulative dose of CT (1 × 10^−7^–3 × 10^−4^ M) was then evaluated on CCh (1 µM)-induced contractions. The concentration-dependent responses obtained were compared with the control group. The relaxant effect was expressed as a percentage of the contraction induced by CCh (1 µM). Concentration-relaxation curves were generated cumulatively.

##### Evaluation of the Effect of Potassium (K+) Channel Activity on Isolated Tracheal Rings

The involvement of K^+^ channels in the bronchodilator effect of CT was assessed by pre-incubating tracheal tissues for 20 min with different K^+^ channel antagonists before CCh (1 µM)-induced contraction. The antagonists used were TEA (1 mM; non-selective K^+^ channel blocker), 4-aminopyridine (4-AP, 100 µM; voltage-dependent K^+^ channel blocker), and glibenclamide (Gb, 1 µM; ATP-sensitive K^+^ channel blocker) [51]. After pre-treatment, cumulative concentrations of CT (1 × 10^−7^–3 × 10^−4^ M) were applied, and their effects on CCh-induced contractions were recorded. Relaxant responses were expressed as percentages of CCh-induced contraction, and concentration–relaxation curves were constructed cumulatively.

### 4.5. Statistical Analysis

When evaluating the effect of cryptotanshinone (CT) on control responses, the contractile responses to carbachol (CCh, 1 µM) or potassium chloride (KCl, 80 mM) were expressed as a percentage of the respective control responses. Statistical analyses were performed using SPSS software (version 25.0; SPSS Inc., Chicago, IL, USA). Differences between two groups were assessed using the Mann–Whitney U test, whereas comparisons among multiple groups were analyzed using the Kruskal–Wallis test followed by Bonferroni-corrected post hoc analysis.

Graphical plots and concentration–response curve fitting were performed using GraphPad Prism (version 8.02; GraphPad Software, San Diego, CA, USA). Concentration–response curves were analyzed by nonlinear regression using a log agonist versus response model. The value of *p* < 0.05 was considered statistically significant. All experimental data are presented as mean ± standard error of the mean (SEM).

## Figures and Tables

**Table 1:** Molecular docking binding affinity scores (kcal/mol; mean ± SD) of cryptotanshinone (CT) against M3 muscarinic receptor and L-type voltage-dependent calcium channel (L-VDCC) targets.

PDB Code	Docking Score (kcal/mol)
4U15 (M3 receptor)	−9.18 ± 0.26
8WE8 (L-type calcium channel)	−8.81 ± 0.94

## Data Availability

The datasets created and/or analyzed during the current study are accessible from the corresponding author upon reasonable request.

## Abbreviations

ACh, acetylcholine; ASM, airway smooth muscle; AA, arachidonic acid; AHR, airway hyperresponsiveness; β_2_-AR, beta-2 adrenergic receptor; BKCa, large-conductance Ca^2+^-activated K^+^ channel; CCh, carbachol; cAMP, cyclic adenosine monophosphate; Ca^2+^, calcium ion; cGMP, cyclic guanosine monophosphate; COX, cyclooxygenase; CRC, concentration–response curve; CT, cryptotanshinone; DAG, diacylglycerol;EDTA, ethylenediaminetetraacetic acid disodium salt (EDTA-Na_2_); GPCR, G protein-coupled receptor; IP_3_, inositol trisphosphate; K^+^, potassium ion; KATP, ATP-sensitive potassium channel; Kv, voltage-gated potassium channel; L-VDCC, L-type voltage-dependent calcium channel; L-NAME, Nω-nitro-L-arginine methyl ester; MLCK, myosin light chain kinase; NO, nitric oxide; NOS, nitric oxide synthase; ODQ, 1H-[1,2,4]oxadiazolo[4,3-a]quinoxalin-1-one; PG, prostaglandin; PKA, protein kinase A; PKC, protein kinase C; PLCβ, phospholipase C beta; TEA, tetraethylammonium.
